# ILC2s: New Actors in Tumor Immunity

**DOI:** 10.3389/fimmu.2019.02801

**Published:** 2019-12-03

**Authors:** Giuseppe Ercolano, Maryline Falquet, Giulia Vanoni, Sara Trabanelli, Camilla Jandus

**Affiliations:** Department of Oncology, Ludwig Institute for Cancer Research Lausanne, Centre Hospitalier Universitaire Vaudois (CHUV), University of Lausanne, Lausanne, Switzerland

**Keywords:** patients, immunotherapy, cancer, ILC2, IL-33, ST2

## Abstract

Innate lymphoid cells (ILCs) represent the most recently identified family of innate lymphocytes that act as first responders, maintaining tissue homeostasis and protecting epithelial barriers. In the last few years, group 2 ILCs (ILC2s) have emerged as key regulators in several immunological processes such as asthma and allergy. Whilst ILC2s are currently being evaluated as novel targets for immunotherapy in these diseases, their involvement in tumor immunity has only recently begun to be deciphered. Here, we provide a comprehensive overview of the pleiotropic roles of ILC2s in different tumor settings. Furthermore, we discuss how different therapeutic approaches targeting ILC2s could improve the efficacy of current tumor immunotherapies.

## Introduction

ILCs are the most recently described family of innate immune cells that play a key role in the preservation of epithelial integrity and tissue immunity (1). ILCs are rapidly activated by both tissue and immune cell-derived signals providing the first line of defense against bacterial, viral and helminthic infections (2–6). However, ILCs need to be tightly regulated, given that their uncontrolled activation and proliferation has been shown to contribute to severe inflammation and damage in gut, lung, skin, and liver (7). ILCs are classified into three different groups, according to the expression of specific transcription factors and surface markers, and based on their cytokine secretion profile (8).

In humans, ILC1s define the T-bet-dependent ILC subset that mainly produce IFNγ and TNFα (9). ILC3s rely on RORγT for their development and express CD117 (also referred to as c-Kit) on their cell surface (10). ILC2s comprise the GATA-3-dependent ILC subset that is also characterized by the expression of the prostaglandin D2 receptor 2 (CRTH2), the IL-33 receptor (IL1RL1 also referred as ST2) and by variable levels of c-Kit (11). More recently, Nagasawa and colleagues showed that the killer cell lectin-like receptor subfamily G member 1 (KLRG1) is a surface marker that arises during ILC2 development in humans (12). KLRG1 is a co-inhibitory receptor already reported to be expressed also by CD4^+^ and CD8^+^ T cells as well as by NK cells, that binds to the members of the cadherin family (13, 14). In mice, the ILC2 phenotype is characterized by the surface expression of both ST2 and KLRG1 (15). Notably, ST2^+^KLRG1^+/−^ ILC2s are defined as natural ILC2s (nILC2s) which respond to IL-33 (15), whilst ST2^−^ KLRG1^hi^ ILC2s represent inflammatory ILC2s (iILC2s) reported to differentiate during infections. iILC2s are highly responsive to IL-25, but not to IL-33, and are able to differentiate into ILC3-like cells under type-17 stimulation, thus defining a distinct subset from nILC2s. ILC2s are able to respond to a wide range of soluble mediators like alarmins [IL-25, IL-33, and thymic stromal lymphopoietin (TSLP)], survival cytokines (such as IL-2, IL-9, and IL-7) and eicosanoids. In addition, ILC2s have been shown also to respond to neuropeptides, including neuromedin U (NMU), vasoactive intestinal peptide (VIP), and calcitonin gene-related peptide (CGRP) (3, 16–20). More precisely, it has been shown that neuropeptides released by pulmonary endocrine cells (PNECs) can stimulate resident ILC2s to produce cytokines, such as IL-5, which in turn support downstream type-2 immune responses (21). Similarly, VIP can stimulate IL-5 release by ILC2s, regulating eosinophil homeostasis in intestinal tissues (22). On the contrary, an opposite role for the CGRP was described, as it can negatively modulate ILC2 effector functions (i.e., cytokine production) in the context of lung inflammation and also during helminth infections (23, 24). It has also been reported that ILC2s in the small intestine, express high levels of the β_2_- adrenergic receptor (β_2_-AR), which acts as a negative regulator of the ILC2-mediated anti-inflammatory response (25).

Once activated, ILC2s secrete type 2 cytokines, such as IL-4, IL-5, IL-9, IL-13, and amphiregulin (AREG), that are involved in airway responses, helminth expulsion, and tissue repair (26). More recently, it has been reported that activated ILC2s are able to produce prostaglandin D2 (PGD2) that acts in an autologous manner supporting ILC2 function via the CRTH2 receptor (27). A detrimental role of ILC2s in chronic inflammation is suggested by their increased frequency in the peripheral blood of asthma and chronic rhinosinusitis patients; and additionally, the secretion of AREG by intrahepatic ILC2s is thought to contribute to the process of fibrogenesis in liver diseases (28, 29).

**Figure 1:**
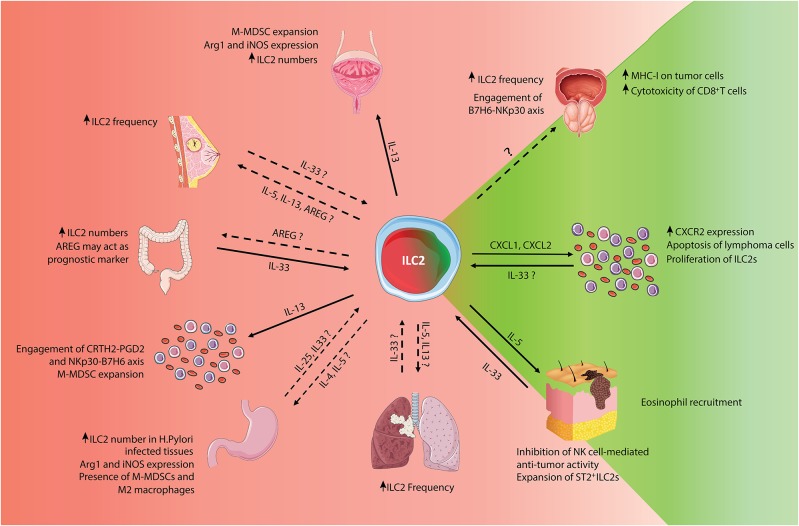
Schematic representation of pro- and anti-tumor roles of ILC2s. Summary of the known pro- and anti-tumor roles of ILC2s, classified by tumor types. For bibliographic details refer to the work cited in the main manuscript.

However, in cancer, the role of ILC2s is still controversial. Elevated numbers of ILC2s have been found in many IL-33-enriched tumors, such as breast, gastric and prostate cancer (30–32) as IL-33 is an ILC2 activator that can promote tumor growth, metastatic dissemination and angiogenesis (33). The ILC2 pro-tumorigenic activity is mainly ascribed to the IL-33-triggered IL-4 and IL-13 production. These cytokines have been reported to support tumor development and progression (34), in part by the recruitment and activation of monocytic myeloid-derived suppressor cells (M-MDSCs) that are considered potent inhibitors of the anti-cancer immune response (35). In addition, AREG produced by ILC2s, can further suppress the anti-tumor immune response by boosting the activity of regulatory T cells (Tregs) (36). Conversely, ILC2-produced IL-5 promotes blood and tissue eosinophilia that correlates with reduced tumorigenicity and tumor progression in mice (37). In this review, we summarize the current knowledge concerning the presence and functional characteristics of ILC2 populations in different tumors, using both patient samples and murine tumor models (Figure 1). Furthermore, we discuss potential strategies to exploit ILC2 biology to improve the efficacy of current tumor immunotherapies.

## ILC2s in Hematological Malignancies

Hematological malignancies represent the fourth most common type of cancer (38). ILCs are a rare cell population, representing ~0.4% of total circulating peripheral blood lymphocytes in humans (39), however, we have reported that ILC2s are expanded in the peripheral blood of acute promyelocytic leukemia (APL) patients at diagnosis, compared to healthy donors. In particular, we found that ILC2s have a central role in the establishment of an immunosuppressive axis, dictated by the tumor-derived factors PGD2 and B7H6 and their ILC2 receptors CRTH2 and NKp30, respectively. This interaction triggers the production of IL-13 which in turn recruits M-MDSCs supporting the growth of cancer cells [(31); Figure 1, left lower corner]. These findings were also confirmed in an APL mouse model raising the possibility of finding the same axis in other tumors, including solid tumors such as prostate cancer (see “*ILC2s in prostate cancer*” section).

In contrast, in treatment-naïve patients with acute myeloid leukemia (AML), we and others have observed an expansion of ILC1s. There was no detection of a change in ILC2 frequency but we observed a lower production of IL-5 and IL-13 following *in vitro* short-term activation with phorbol 12-myristate 13-acetate (PMA) plus ionomycin (40). In this context, the increased ILC1 frequency might be due to the conversion of ILC3s and/or ILC2s into ILC1s driven by tumor-derived factors, among others TGFβ. A putative anti-tumor role of ILC2s has been proposed in a subcutaneous lymphoma mouse model, where sustained production of IL-33 induced the upregulation of CXCR2 on EL4 thymoma cells, the expansion of ILC2s and the concomitant production of CXCR2 ligands (CXCLs). These ligands, mainly CXCL1 and CXCL2, induced apoptosis in a limited proportion of lymphoma cells, thus limiting tumor progression [(41); Figure 1, right middle panel].

## ILC2s in Urogenital Tract Cancers

### ILC2s in Prostate Cancer

Prostate cancer is the most common non-cutaneous malignancy in men and responsible for about 20% of male cancer-related deaths (42). Despite the different therapeutic approaches, including the use of immune checkpoint inhibitors, limited clinical benefits have been observed in patients (43). In this context, the tumor microenvironment (TME) seems to play a key role in driving prostate cancer progression and chemoresistance (44, 45). Focusing on ILCs in prostate cancer patients, we have shown that ILC2 levels positively correlate with tumor stage and with M-MDSC frequency (31). Additionally, DU145 and PC3 prostate cancer cells secrete the ILC2 activator PGD2 and express high levels of B7H6, the ligand of NKp30, corresponding with the immunosuppressive axis found in APL patients. Using the spontaneous TRAMP model, in which mice develop orthotopic prostate tumors from puberty (46), we observed an increase of ILC2s both in the blood and the tumor supporting our findings in prostate cancer patients (31). Conversely, Saranchova et al. have showed that ILC2s can acquire anti-tumor activities by influencing the effector functions of cytotoxic lymphocytes, through the release of IL-5 and IL-13 acting on DCs. They used the pTAP-1-EGFP-stably-transfected LMD cell line, derived from a metastatic prostate cancer mouse model, in which TAP-1 activation in tumor cells indirectly correlates with MHC-I and EGFP expression. In order to mimic metastatic prostate cancer conditions *in vivo*, the authors isolated ILC2s from tumors of donor mice and cultured them with the LMD cell lines, CD8^+^ dendritic cells (DCs), ovalbumin (OVA) peptide as well as CD8^+^ OT-1 T cells. They observed an increased expression of EGFP, indicating that the LMD cells had been stimulated to express MHC-I on their surface by ILC2s. This suggests that ILC2s via direct interaction or cytokine secretion, facilitate antigen presentation and the recognition of tumor cells by T cells, thus improving the anti-tumor adaptive response [(47); Figure 1, right upper panel]. The use of tumor-bearing ILC2 KO mice transferred with ILC2s isolated from wild-type donor mice might represent a good strategy to dissect the contribution of ILC2s in the T-cell mediated anti-tumor responses.

### ILC2s in Bladder Cancer

Bladder cancer (BC) is broadly divided into two major stages: non-muscle-invasive (NMIBC) and muscle-invasive bladder cancer (MIBC) and is the ninth most common cancer worldwide (48). NMIBC standard treatment involves intravesical instillation of the Bacillus Calmette-Guérin antigen (BCG) (49), whereas MIBC treatment involves neoadjuvant cisplatin-based chemotherapy (NAC) followed by radical cystectomy (50). Despite these approaches, the rate of recurrence of BC remains high (51). To better understand the reasons behind BC treatment failure, immune cell distribution was analyzed in the urine of NMIBC patients during BCG treatment and ILC2s were found to be the most abundant innate lymphoid cell subpopulation present (52). ILC2 frequency positively correlates with M-MDSC frequency but not with T cell numbers suggesting that ILC2s may promote the expansion of M-MDSCs. This correlation has also been confirmed in the blood of patients with MIBC. In addition, ILC2-associated cytokines measured in blood and urine samples of NMIBC and MBIC patients showed a significantly elevated level of IL-13 compared to healthy donors. IL-13 secretion could explain the ILC2-dependent recruitment of M-MDCS which were shown to express the IL-13 receptor α1 (IL-13Rα1). At mRNA level, the immunosuppressive properties of IL-13 were demonstrated with upregulation of monocytic suppressive markers such as arginase 1 (Arg1), inducible nitric oxide synthase (iNOS) and C/EBPβ [(52); Figure 1, upper central panel]. These results highlight the concept that the BC immunosuppressive environment is, at least in part, driven by ILC2-derived IL-13 that may be contributing to the failure of current BC therapies. Furthermore, the ratio between T cells and M-MDSCs may also have an impact on the response to treatment, since patients with a high T cell/MDSC ratio show improved survival with reduced risk of recurrence. However, more research is needed to better understand the role of ILC2s in this type of cancer.

## ILC2s in Cancers of the Gastrointestinal System

### ILC2s in Colorectal Cancer

Colorectal cancer (CRC) is the third and second most common cancer diagnosed in men and women, respectively (53). CRC mortality rate has decreased over recent years due to improved cancer screening methods (54). A variety of genetic, environmental and nutritional factors play a key role in the pathogenesis and progression of CRC (55). Several immune cell populations infiltrate the CRC TME by modulating the tumor response (56). Among them, ILC2s, that are abundant in the intestinal mucosa (57), have been reported in CRC patients, to be recruited to the tumor site suggesting their potential role in CRC development and progression (58). However, there is still no robust data in human or mouse models, clarifying the role of ILC2s in colorectal tumorigenesis. Nevertheless, analysis of human resected CRC specimens has shown that SW480 and SW620 cells at different stages of the disease are positive for IL-33 and its receptor ST2 (59–61). IL-33 has been shown to promote the *in vitro* proliferation of freshly isolated primary CRC cells (the HT-29 CRC cell line and the murine MC38 cell line), through the activation of the ST2 receptor. The IL-33/ST2 axis activates NF-kB signaling which in turn induces cyclooxygenase-2 (COX2) expression and prostaglandin E2 (PGE_2_) synthesis, triggering CRC cell proliferation (62). Further evidence for involvement of the IL-33/ST2 axis in CRC pathogenesis comes from an inflammation-driven model in which ST2 deficiency in mice conferred protection against tumor development (61) and secondly from a polyposis mouse model (Apc^Min/+^), where abrogation of IL-33 signaling reduced the tumor burden, Th2-associated cytokine production and mast cell activation (59). Conversely, Akimoto et al. have reported that sST2, a soluble form of the IL-33 receptor, is down-regulated in patient serum and correlates inversely with disease progression. This data has also been confirmed in nude mice, in which injection of short hairpin RNA (shRNA) targeting sST2, triggered tumor development, and progression (60). These findings underline the potential dual role of the IL-33/ST2 axis in colon cancer (63) and the need for further analysis of this pathway in different CRC models. AREG is another important molecule that regulates cancer cell proliferation, invasion and angiogenesis (64) and has been proposed as a prognostic marker in CRC (65). AREG upregulation is associated with increased migration and invasion of CRC cells which is essential for metastasis [(66, 67); Figure 1, left middle panel]. AREG can be produced by different immune cell types under pro-inflammatory conditions, such as mast cells, basophils, tissue resident CD4 T cells (68). However, no data is available to date on ILC2-derived AREG in CRC development and progression.

### ILC2s in Gastric Cancer

With a 65% overall survival rate, gastric cancer is one of the most common malignancies affecting the digestive system, with more than one million people newly diagnosed each year worldwide (69). However, due to poor population strategies for primary prevention and lack of early symptoms, most patients are diagnosed at an advanced stage with limited benefit from existing therapies (70). The use of immunotherapy for the treatment of metastatic gastric cancer such as pembrolizumab has showed promising effects in Phase I clinical trials (71), but other strategies are still needed to improve patient survival. Gastric tumors are multifactorial in etiology and one of the main risk factors for disease is chronic infection with Helicobacter Pylori *(H. Pylori*) (72). *H. Pylori* infection causes chronic inflammation of gastric tissue, favoring the development of gastric carcinoma (73). Higher numbers of ILC2s have been observed in the tumors of gastric cancer patients infected with *H. Pylori*, suggesting a role for ILC2s in this immunosuppressive type 2 environment [(74); Figure 1, left lower panel]. Moreover, the frequency of ILC2s in the peripheral blood mononuclear cell (PBMC) compartment is higher in gastric cancer patients than in healthy volunteers and ILC2-associated cytokines, such as IL-4, IL-5, and IL-13, are increased in gastric cancer patients, both at mRNA and protein level in PBMCs and plasma, respectively. In addition, Arg1 and iNOS, expressed in M-MDSCs and M2 macrophages as well as in group 2 ILCs (75, 76) were found to be highly expressed at mRNA level in PBMCs of gastric cancer patients (77). Moreover, type 2 cytokines derived from ILC2s have been reported to mediate Arg1 and iNOS secretion by MDSCs and M2 macrophages suggesting a role for ILC2s in promoting M-MDSCs and M2 macrophage phenotype and favoring their immunosuppressive function (78, 79). However, using the gp130^FF^ mouse model, validated as a model of spontaneous gastric cancer, Eissmann et al. (80), demonstrate that mast cells, rather than ILC2s, promote tumor growth upon IL-33 stimulation. The authors show that mast cells are more abundant than ILC2s in gastric tumors and secrete macrophage-chemoattractant colony-stimulating factor 2 (CSF2), CCL3, and IL-6 in response to activation by tumor-derived IL-33. In ST2 deficient animals (gp130^FF^ ST2^−/−^ mice), the authors observed lower tumor burden, which was increased upon adoptive transfer of ST2^+^ wild type bone marrow-derived mast cells (BMMC). Therefore, additional studies with adoptive transfer of ST2^+^ wild type ILC2s could help to determine the individual contribution of mast cells and ILC2s in this cancer setting.

## ILC2s in Breast Cancer

Breast cancer is the most common cancer affecting women and its incidence rate in younger women is expected to increase (81). Despite the progress in breast cancer detection and treatment (82), aggressive tumors, such as triple negative breast cancer (TNBC), still lack targeted therapies (83). Immunotherapeutic strategies provide hope of finding new treatment approaches (84), but due to the high heterogeneity of breast cancer (85), much more needs to be done to fully understand the interactions between immune and breast cancer cells (86). ILC2 frequency has been shown to be higher in malignant compared to benign breast tissue in humans (32). Using the 4T1 mammary carcinoma model, Jovanovic et al. have reported an increase in endogenous levels of IL-33, that correlated with cancer progression and metastasis. Using the parental 4T1 cell line overexpressing IL-33, they showed elevated frequencies of IL-5 and IL-13-expressing ILCs in tumor-bearing mice [(33); Figure 1, left upper panel]. More precisely, in this model they found that ILC2s trigger tumor progression and metastasis development in response to IL-33, sustaining the immunosuppressive milieu that characterizes breast cancer patients. This data suggests that ILC2s could be activated by IL-33 to secrete IL-5 and IL-13 in the 4T1 model of breast cancer, but further investigation is required to confirm this finding also in patients. Moreover, it has been shown that AREG regulates the proliferation and the migration of different mouse and human estrogen-receptor positive (ER2^+^) breast cancer cell lines (87). However, it is still unknown whether ILC2s and ILC2-derived AREG are involved in this pro-tumoral axis. The use of ILC2 KO mice could represent a strategy to address the role of AREG-producing ILC2s in the context of breast cancer.

## ILC2s in Melanoma

Melanoma is the most aggressive form of skin cancer with a high mortality rate (88). Whilst early stage melanoma is usually curable with surgery, metastatic melanoma is difficult to treat and often fatal. Nevertheless, in the last few years, treatment for metastatic melanoma has advanced due to the introduction of cytotoxic T-lymphocyte-associated antigen-4 (CTLA-4) and the programmed cell-death protein 1 (PD-1) checkpoint inhibitors (89). However, despite these promising discoveries, a high percentage of patients still experience treatment resistance (90) emphasizing the need to find new therapeutic approaches. The TME has been identified recently as a potential target for metastatic melanoma immunotherapy (91). Among the different TME mediators, IL-33 has been reported to inhibit tumor growth in a melanoma mouse model, by stimulating the anti-tumor activity of CD8^+^ T cells and natural killer (NK) cells (92). However, this cytokine has also been shown to bind to and expand ST2^+^ tumor-infiltrating ILC2s, characterized by the expression of the immunosuppressive ectoenzyme CD73. In this setting, ILC2s partially antagonized the IL-33 dependent, NK cell-mediated anti-tumor response, as evidenced by cell depleting experiments in which the lack of ILC2 CD73^+^ cells led to enhanced NK cell activity and better tumor control (92). This data shows that IL-33 has both a beneficial anti-tumoral role via adaptive immune cells but also a pro-tumoral role via ILC2s. IL-33 is also able to stimulate ILC2s to produce IL-5, a potent eosinophil chemoattractant. Ikutani et al. showed that, in a murine model of metastatic melanoma, the main source of IL-5 was a CD3^neg^ population, characterized by the expression of CD90, CD127, CD25, and ST2 (*bona fide* ILC2s). IL-5 was crucial to induce tumor rejection via eosinophil recruitment, also resulting in reduced lung metastases [(93); Figure 1, right lower panel]. The use of neutralizing antibodies directed against IL-5 may be useful to confirm the involvement of ILC2s in metastatic melanoma.

## ILC2s in Lung Cancer

Lung cancer is generally divided into two types, small cell lung cancer (SCLC) and non-small cell lung cancer (NSCLC) (94). It is strongly correlated with cigarette smoking (95, 96) and is the most common cause of cancer-related deaths. Different targeted immunotherapies are now being used in lung cancer patients including anti-PD-1 antibodies that have been recently approved for the treatment of SCLC (97–99). Nonetheless, a significant percentage of patients do not respond or develop resistance to treatment, leading to consequent cancer progression (96, 100, 101). ILC2s constitute the most prominent ILC subset in the respiratory tract under physiologic conditions, although their overall numbers are low (26). They respond rapidly to tissue-derived alarmins (102), therefore, unsurprisingly, circulating ILC2s and M-MDSCs were found to be increased in a cohort of 36 lung cancer patients at diagnosis and correlated with a strong type 2 phenotype (103). The expansion of ILC2s in the periphery was accompanied by higher levels of IL-5, IL-13, IL-33, and Arg1 in the plasma of lung cancer patients compared to healthy donors. Simoni et al. have also detected ILC2s within lung tumor tissues. However, no functional assays were performed in these studies to define the pro- or anti-tumor roles of ILC2s in lung cancer [(58); Figure 1, central lower panel]. It can be speculated that the observed strong type 2 phenotype may represent a targetable axis for the development of new immunotherapeutic strategies, fostering the anti-tumor immune response. In contrast to these observations, Carrega et al., reported a reduced frequency of ILC2s in tumors compared to normal lung tissue (104). However, in the absence of sufficient data on the function of ILC2s in lung cancer, it is too early to define their role in this setting.

## Future Perspectives and Concluding Remarks

Tumorigenesis is the result of multiple cell intrinsic (e.g., uncontrolled proliferation, cell migration) and cell extrinsic (e.g., pro-inflammatory or immunosuppressive microenvironment, growth factors, angiogenesis) factors (105). Among the latter, the contribution of the immune system to tumor development and/or tumor cell clearance has become more and more accepted/relevant (106). Even though the impact of ILC2s in malignancy is not currently well defined, the number of studies focusing on the role of ILC2s in tumor immunity has multiplied (107), highlighting the importance of this cell type during cancer development and progression. However, many aspects still need to be elucidated to achieve a better understanding of the mechanisms behind ILC2 pro- and anti-tumoral functions. Moreover, it is known that ILC2s are highly plastic cells that can easily adapt to the environment to which they are exposed (12). Hence, the cytokines present in the TME may stimulate the conversion of ILC2s into other ILC subsets within the tumor tissues, suggesting that the environment that they are exposed to can dictate their pro- and/or anti-tumoral roles. In the nasal polyps of cystic fibrosis patients, ILC2s are reported to be capable of differentiation into IL-17 producing cells when stimulated with IL-1β, IL-23, and TGF-β, the concomitant downregulation of GATA-3 and increased expression of RORγt were also observed (108). Therefore, ILC2s may be detrimental in the pathogenesis of IL-17-associated diseases, including some types of cancer. Efforts to understand the role of *bona fide* and/or plastic ILC2s in tumors represents the next challenging step. In this endeavor, the use of mouse models will be crucial, for example, the use of genetically engineered ILC2-depleted mice would allow dissection of the real contribution of this cell type to tumor development and/or progression (109). However, ILC2 characterization at any given time point in tumor-bearing mice will always be difficult due to their inherent plasticity. Moreover, the contribution of nILC2s and iILC2s remains to be elucidated in the tumor setting. Given their different abilities to respond to cytokines and, therefore, their potential distinct pro- and/or anti-tumor roles, further investigation should consider both subsets separately. The use of reporter mice, such as the Il13^GFP^ or other type-2 cytokine reporter animals, represent helpful tools to track ILC2s, independently of their transcriptional profiles that can be shaped by the interaction with tumor cells and/or by the TME. The use of humanized mice (BRGST HIS mice) to establish patient-derived xenograft (PDX) models would provide unique environments for interrogation of the function of the innate immune system, in particular, the contribution of ILC2s to cancer development and progression (110). Collectively, these strategies are expected to accelerate our knowledge of ILC2 biology, and provide new insight into potential therapeutic targets. One approach may be to target Th2-associated cytokines and ILC2-secreted molecules using neutralizing antibodies. This is the same technique employed by some NK cell-based immunotherapies, for example the use of a transforming growth factor beta (TGF-β) antibody to block TGF-β signaling, restores NK anti-tumor activity and synergy with α-PD-1 can be achieved (111). These anti-cytokine-based immunotherapies may also be effective for altering ILC2 function. However, they are a challenging and non-specific target due to the diversity of cell types producing them and their multiple roles in different physiological and pathophysiological processes. Another attractive strategy for targeting ILC2s may involve the disruption of transcription factor signatures, that are emerging as indispensable in ILC2 biology, or the manipulation of their metabolic programs. Lastly, remarkable success has been recently achieved in the clinics by immunotherapy based on immune checkpoint blockade, including agents targeting CTLA4, PD-1, or PD-L1. While the pattern of CTLA4 and PD-1 expression in various subsets of CD4^+^ T and CD8^+^ T cells are well-understood, little is known on the expression of immune checkpoints in ILCs. Of note, PD-1 has been reported as an intrinsic negative regulator of the functions of the ILC2 subset in mice, raising the possibility that current treatments targeting PD-1 might significantly impact on ILC functions (112). Ultimately with constant new discoveries in the ILC2 field in health and disease, immunotherapies focusing on the functional targeting of ILC2 are fast approaching clinical realization.

## Author Contributions

GE, MF, and GV wrote the manuscript. ST and CJ wrote and critically revised the manuscript. All authors provided approval for publication of the manuscript.

### Conflict of Interest

The authors declare that the research was conducted in the absence of any commercial or financial relationships that could be construed as a potential conflict of interest.
